# Bio-Guided Fractionation of Ethanol Extract of Leaves of *Esenbeckia alata* Kunt (Rutaceae) Led to the Isolation of Two Cytotoxic Quinoline Alkaloids: Evidence of Selectivity Against Leukemia Cells

**DOI:** 10.3390/biom9100585

**Published:** 2019-10-08

**Authors:** Juan Manuel Álvarez-Caballero, Luis Enrique Cuca-Suárez, Ericsson Coy-Barrera

**Affiliations:** 1 Grupo de Química y Bioprospección de Productos Naturales, Universidad del Magdalena, Santa Marta 470004, Colombia; 2 Laboratorio de Investigación en Productos Naturales Vegetales, Facultad de Ciencias, Departamento de Química, Universidad Nacional de Colombia, Bogotá D.C. 111321, Colombia; 3 Bioorganic Chemistry Laboratory, Facultad de Ciencias Básicas y Aplicadas, Universidad Militar Nueva Granada, Cajicá 250247, Colombia; ericsson.coy@unimilitar.edu.co

**Keywords:** cytotoxicity, Rutaceae, *Esenbeckia alata*, bio-guided fractionation, alkaloids, ubiquitin-activating enzyme 5

## Abstract

Bio-guided fractionation performed on the leaves-derived ethanol extract of *Esenbeckia alata* (Rutaceae), a plant used in traditional medicine, led to the isolation of two alkaloids, kokusaginine **1** and flindersiamine **2**, as main cytotoxic agents. Primary ethanolic extract and raw fractions exhibited cell inhibition against five cancer cell lines at different levels (25–97% inhibition at 50 µg/mL) as well as isolated alkaloids **1**–**2** (30–90% inhibition at 20 µM). Although alkaloid **2** generally was the most active compound, both alkaloids showed a selective effect on K562, a human chronic myelogenous leukemia cell line. The E1-like ubiquitin-activating enzymes (e.g., UBA5) have been recently described as important targets for future treatment of cancer progression, such as leukemia, among others. Therefore, as a rationale to the observed cytotoxic selectivity, an in-silico evaluation by molecular docking and molecular dynamics was also explored. Compounds **1**–**2** exhibited good performance on the interaction within the active site of UBA5.

## 1. Introduction

One of the most-studied botanical families, due to its many uses in traditional medicine, is the Rutaceae family [1,2]. It has around 1600 species gathered in 160 genera, distributed in temperate and tropical zones of both hemispheres [3]. Genus *Esenbeckia* is one of the most abundant taxonomic group within this family, contributing a total of 30 species [4]. There are several reports linking the use of plants from this taxon in folk practices because of their medicinal properties. For instance, leaves and roots of *E. yaxhoob* are used by the Mexican people for the treatment of gastrointestinal diseases, epilepsy, headaches, and as an antidiuretic agent [5,6] as well as *E. alata*, *E. panamensis*, and *E. pentaphylla* as insecticide, antispasmodic, and dermatological [7,8]. In particular, the aerial part of the medicinal shrub *Esenbeckia alata* is used as a febrifuge and insecticide in the Atlantic Coast of Colombia [9], promoting a particular interest for this plant. In previous phytochemical studies performed on *E. alata*, four metabolites (identified as 5-hydroxy-2-methylchromanone, (-)-episesamine, pellitonin, and sitosterol) were isolated from bark [9], whereas furanocoumarins, pyranocumarins, lignans, and quinoline alkaloids were obtained from leaves [10,11]. From this set of isolated metabolites, quinoline alkaloids are considered as taxonomic markers of the genus and they have been identified in several *Esenbeckia* plants [4,12,13,14,15]. In addition to this chemotaxonomic value, quinoline alkaloids deserve importance due to the wide variety of activities they have shown [16] and cytotoxic activity can be particularly highlighted [17,18,19].

Under these facts, and as part of our search for bioactive substances from Rutaceae plants, a bio-guided fractionation (based on a selection related to the cytotoxic activity against six cancer cell lines such as U251, PC-3, K562, HCT-15, MCF-7, and SKLU-1) led to the isolation of two main known alkaloids (flindersiamine **1** and kokusaginine **2**) from ethanol extract of *E. alata* leaves. Owing to alkaloids **1**–**2** showing selectivity towards inhibition against K562, a human chronic myelogenous leukemia cell line which exhibits sensitivity to ubiquitin-like system perturbations, the study was deepened towards the in-silico interaction of test alkaloids with a component of such a system. The rationale is based on the fact that E1-like ubiquitin-activating related enzymes (UAE) have been described as important targets for future treatment of cancer, especially leukemia myeloma [20]. UAE participates in the ubiquitylation cascade within the ubiquitin proteasome system (UPS) [21], so the inhibition of UAE E1 resulted in low abundance of ubiquitinated proteins in leukemia and myeloma cells, inducing cell death [20,22]. Ubiquitination is the central process in UPS for regulating protein degradation, subcellular localization, and cell cycle progression [23]. This process is a three-step enzymatic cascade (E1 (activating), E2 (conjugating), E3 (ligating)) where target proteins are labeled with ubiquitin (Ub) and ubiquitin-like (Ubl) small protein modifiers [24]. Nine Ub/Ubl modifiers are currently known. Among them, the Ub-fold modifier 1 (UFM1) [25] is associated with several important functions [26], even cancer [27]. The first process within the three-step cascade (called the ufmylation pathway), the UFM1-activating enzyme E1 (a class of UAE E1 called UBA5), catalyzes the ATP-dependent adenylation of the UFM1 *C*-terminal carboxylate [28]. Since UBA5 is very important for the UFM1 protein conjugation in erythroid development and for promoting proliferation and survival of cancer cells, this enzyme is considered as a therapeutic target for disrupting cancer progression [29]. Thus, in order to rationalize the observed cytotoxic selectivity, an in-silico evaluation through molecular docking and molecular dynamics was then used as an approach to provide computational hypotheses related to describe some insights into the interaction of test alkaloids **1**–**2** and the active site of the UBA5 enzyme. This work constitutes the first report of cytotoxic activity on this set of test cell lines for the *E. alata* plant and the cytotoxic alkaloids **1**–**2.**

## 2. Experimental Section

### 2.1. Plant Material

The plant sample, corresponding to leaves of *Esenbeckia alata* Kunt. (Rutaceae), was collected in Montes de María, Department of Bolívar, Colombia. The specimen was identified by the botanist Eduino Carbonó and a voucher was deposited in the Herbarium of the University of Magdalena with the collection number 001 (UTMC).

### 2.2. Bio-Guided Fractionation

**Figure 1:**
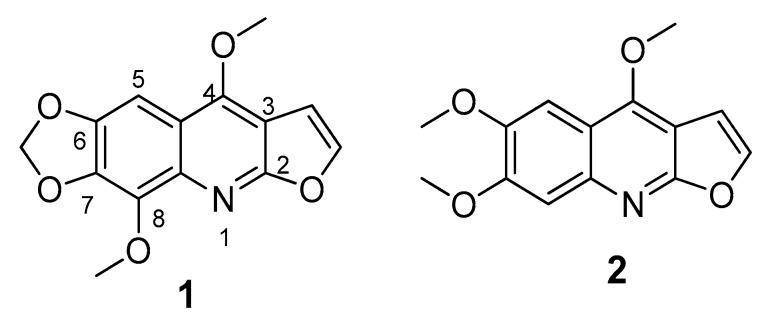
Furoquinoline alkaloids, flindersiamine **1**, and kokusaginine **2**, isolated from leaves of *E. alata*.

The same previously reported extract from leaves of *E. alata* [11] was used for the present cytotoxicity-guided fractionation study. A portion of this primary ethanol extract (called EEa, 65 g) was fractionated by vacuum column chromatography (VCC) (80 × 8 cm) on silica gel using different solvents by increasing polarity, in order to have several fractions eluted with *n*-hexane (5.4 g), ethyl acetate (EtOAC) (2.3 g), methanol (MeOH) (25.6 g), and water (25.5 g). All resulting fractions were evaluated on cytotoxic activity. The most active fraction (EtOAc called EA-EEa, 1515 mg) was depurated by column chromatography (CC) on silica gel using chloroform/MeOH (8:2) (3.0 L) as eluent by increasing polarity to collect eight subfractions (EA-EEa1–EA-EEa8). All resulting subfractions were also evaluated on cytotoxic activity. Most active subfractions [EA-EEa3 (235 mg) and EA-EEa6 (195 mg)] were then depurated by CC using dichloromethane/acetone 9:1 (1.5 L) as eluent. Subfraction EA-EEa3 afforded **2** (135 mg) and EA-EEa6 yielded **1** (95 mg) as main compounds retaining the cytotoxic activity exhibited by extracts, fractions, and subfractions. These compounds were structurally elucidated by ^1^H and ^13^C-NMR (see Supplementary Materials) on comparing with authentic compounds previously isolated, whose structures are exposed in Figure 1 [11].

### 2.3. Cytotoxic Activity Test

The cytotoxicity of test substances (ethanol extract, fractions, subfractions, and alkaloids **1**–**2**) was evaluated using a previously reported protocol [30]. Thus, the inhibition assays were separately determined after the treatment on cancer cells (U251 (central nervous system), PC-3 (prostate), K562 (leukemia), HCT-15 (colon), MCF-7 (breast), and SKLU-1 (lung), provided by the National Cancer Institute (NCI), Bethesda, MD, USA) with the ethanol extract of *E. alata* leaves, fractions, subfractions, and the alkaloids **1**–**2**, dissolved in dimethylsulfoxide (DMSO) and diluted in the medium, without exceeding the maximal amount of DMSO (<0.5%). Doxorubicin (10 µM) (Sigma-Aldrich, Milwaukee, WI, USA) was used as a positive control. 100 μL of a solution of the ethanolic extract (50 μg/mL), fractions (30 μg/mL), and subfractions (10 μg/mL) and alkaloids **1**–**2** (20 μM) were added to each cell culture (100 μL). The mixture was kept under exposure for 48 h. After the incubation period, the cells were fixed to the plastic bottom layer by the addition of 50 μL of cold 50% aqueous trichloroacetic acid. The plates were incubated at 4 °C for 1 h, washed with H_2_O, and air dried. The cells with trichloroacetic acid were stained by addition of sulforhodamine B (SRB) (Sigma-Aldrich, Milwaukee, WI, USA) at 0.4% (*w*/*v*) dissolved in 1% acetic acid for 30 min and then washed with 1% aqueous acetic acid. The plates were air dried and the dye was solubilized by adding 100 μL of Tris, pH 10. The plates were shaken for 5 min and the absorbance was measured by an ELISA plate reader (Bio-Tex instruments, Winooski, VT, USA) at 515 nm. These values were expressed as an inhibition percentage, relative to the incubations treated with negative control.

### 2.4. Molecular Docking

A conformational random search using Merck molecular force field (MMFF), without geometric restrictions, included in SPARTAN software (Spartan 14v114 (2013), Wave function, Inc., Irvine, CA, USA) was performed with a 500-conformers limit. Energetically most-stable conformers were MMFF-optimized to be used into the docking protocol. Docking calculations were performed with the Autodock/Vina 1.1.2 (The Scripps Research Institute, La Jolla, CA, USA) plug-in for PyMOL 1.3r2 (Delano Scientific LLC, South San Francisco, CA, USA) under a Python 2.5.2 (Python Software Foundation, Wilmington, DE, USA) environment for Windows [31]. The structure of the UBA5 enzyme was retrieved from the protein data bank RCSB (PDB Code: 3GUC). Docking calculations were then performed between the minimized ligand through a cube (dimensions 24 × 24 × 24 Å, grid spacing 0.375 Å) located in the geometric center (coordinates −32.1, 23.8, −19.1) of the UBA5 active site containing its ATP pocket. Residues Glu209 and Asp183 were involved in this grid box, since they has been exposed to be implicated in Ub/Ubl adenylation [29]. Docking poses were classified according to their docking (Vina) scores (in kJ/mol). Ten replicates were performed for each molecular docking calculation. A known UAE inhibitor, TAK-243, was used as control. 2D-residual interaction diagrams were created and visualized on Discovery Studio 2016 Visualizer Client (Biovia, San Diego, CA, USA).

### 2.5. Molecular Dynamics Simulations

Molecular dynamics (MD) simulations were run in Gromacs 5.0.5 (Stockholm University, Stockholm, Sweden) on an Ubuntu 12.04 server (Canonical Ltd, London, UK). The ligand best pose from docking and the crystal structure of enzyme were employed as input for dynamics simulations of complexes and stand-alone proteins, respectively. Ligands were prepared by adding hydrogen atoms and the corresponding charges using the AM1-BCC charge scheme in UCSF Chimera (University of California, San Fransico, CA, USA). Subsequently, ligand topologies were generated automatically with ACPYPE script. Protein topologies were obtained in Gromacs using the Amber 99SB force field and TIP3P water model was implemented. Solvation was performed in a triclinic box using a margin distance of 1.0 nm. The addition of 0.1 M NaCl to complexes and proteins was carried out by replacing randomly water molecules until neutrality was achieved. The systems were energy-minimized by 2000 steps of the steepest descent method. NVT equilibration at 310 K for 50 ps, followed by NPT equilibration during 500 ps using the Parrinello–Rahman method at 1 bar as reference were conducted on systems using position restraints. Finally, solute position restraints were released and a production run for 150 ns was performed. The temperature and pressure were kept constant at 310 K and 1 bar, respectively. Coordinates were recorded in a 1 fs time step. Electrostatic forces were calculated using the particle-mesh Ewald (PME) method. Periodic boundary conditions were used in all simulations and covalent bond lengths were constrained by the LINCS algorithm.

### 2.6. Binding Free Energy Calculation

Binding free energy was calculated for test alkaloids **1**–**2** and TAK-243 compounds using the molecular mechanic/Poisson-Boltzmann surface area (MM/PBSA) method [32]. Snapshots were then taken from the trajectory files obtained from previous MD simulations and used for calculating the average binding free energy. ΔG_binding_ was calculated using the Lennard-Jones and Coulomb potential. The binding energy calculations were performed for 200 snapshots taken at an interval of 5 ns during the last stable 50 ns period of MD trajectory, using the g_mmpbsa tool of GROMACS, based on MM/PBSA technique [33]. Thus, the binding associations between protein and ligand by decomposing the total binding free energy into each residue were also assessed.

## 3. Results and Discussion

Table 1 shows the results of the cytotoxic activity of primary ethanol extract, AcOEt fraction, subfractions, and alkaloids **1**–**2** against the cancer cell lines U251 (central nervous system), PC-3 (prostate), K562 (leukemia), HCT-15 (colon), MCF-7 (breast), and SKLU-1 (lung) expressed as cell growth inhibition percentages. Test substances showed effects at different levels on the cancer cell lines evaluated at the test doses, but they also exhibited lower cytotoxicity (4–21%) against the cell line MCR5 (normal human lung fibroblasts). A clear selectivity towards K562 cell line was observed. Thus, the ethanolic extract (called EEa) showed values above 60% at 50 μg/mL against the lines K562, PC-3, MCF-7, and SKLU-1, although the inhibition percentage of K562 was particularly found to be the highest (~97%), so a clear selectivity on this cell line was observed. Such percentages were noticed to be similar (and even better) to that of positive control, whose inhibition values were located into the 82–99% range. In contrast, the ethanolic extract showed a low inhibition percentage for lines U251 and HCT-15 (~27–43%).

Once the primary ethanol extract was fractionated, the resulting four raw fractions (*n*-hexane, EtOAC, MeOH, and water) were also evaluated for their cytotoxic activity on the same panel of cell lines. Three of these fractions (i.e., *n*-hexane, MeOH, and water) resulted in inhibition values below 30% (data not shown), so they were discarded for further depurating processing. However, AcOEt fraction (called EA-EEa) exhibited values between 27 and 97%, involving a similar profile to that of EEa, which indicated retention of the initial cytotoxic activity (Table 1). This EA-EEa fraction was then depurated by traditional CC on silica gel, affording eight subfractions, and six of them exhibited low cytotoxicity (data not shown), excepting the subfractions EA-EEa3 and EA-EEa6. These subfractions retained the cytotoxic activity (30–90%) at a similar level (even better) to that of their parent ethanol extract and raw fraction (Table 1). The main components of these subfractions were alkaloid **2** and **1**, respectively, which were subsequently purified to be evaluated as isolated compounds.

In the case of pure alkaloids **1**–**2**, the behavior at 20 μM was also very similar to that of their parent extract, fraction, and subfractions (Table 1). Such compounds showed relevant activity against K562 line (86–93%), moderate for all other cancer cell lines (30–66%), and very low on MRC5 (4–5%). This fact particularly indicated selective activity of **1**–**2** on particular cancer cell lines. Alkaloid **2** was found to be slightly more active, excepting for line SKLU-1, where alkaloid **1** showed a slightly higher effect than alkaloid **2**. Although the inhibition percentages for the lines U251, MCF7, and SKLU-1 are very similar, the results also revealed marked selectivity towards the inhibition of the K562 line, showing a significant effect at the test dose comparable to that of control (Table 1). Alkaloid **2** was previously evaluated against A549 cell line (lung carcinoma) exhibiting moderate cytotoxic activity (IC_50_ = 87.4 μg/mL) [34] as well as a panel of nine drug sensitive and resistant cancer cells, which was assessed by resazurin method [35]. Recently, an isomer of **2**, kokusaginine B, also exhibited no cytotoxic on the same panel [36]. In addition, both alkaloids **1**–**2** exhibited weak cytotoxicity against A2780 human ovarian cancer cell line [37]. However, compound **2** displayed potent inhibitory effect on MCF-7 (breast cancer) by the inhibition of tubulin assembly [38]. These reported results in combination with our results corroborated those previously findings on similar furoquinoline alkaloids regarding the selective cytotoxic activity against particular cell lines [17,39]. In the present study, alkaloids **1**–**2** exhibited selectivity against K562, being the first report of the cytotoxicity assessment of these alkaloids against such a cell line.

As mentioned above, K562 cancer cell line exhibits sensitivity to UPS perturbations, since this system is crucial for regulating protein degradation, subcellular localization, and cell cycle progression. The Ub-fold modifier 1 (UFM1) is particularly associated with several important biological functions [26]. The first process within the three-step ufmylation pathway is mediated by the UFM1-activating enzyme (a class of non-canonical E1-like enzyme called UBA5), which catalyzes the ATP-dependent adenylation of the UFM1 *C*-terminal carboxylate. Therefore, the inhibition of UBA5, through this ATP pocket, can result in low abundance of ubiquitinated proteins in leukemia and myeloma cells, inducing cell death [40]. Owing to this reason, this enzyme is considered as a therapeutic target for disrupting progression of some type of cancer cells [29]. The discovery and development of this kind of inhibitors are very important, since they might be used alone or act synergistically with kinase inhibitors to induce apoptosis of K562 cells [41]. Therefore, the study was then oriented towards the in-silico interaction of test alkaloids **1**–**2** within the active site containing the ATP pocket of UBA5, in order to evaluate their plausible competing ability for this site acting as putative inhibitors. 

**Figure 2:**
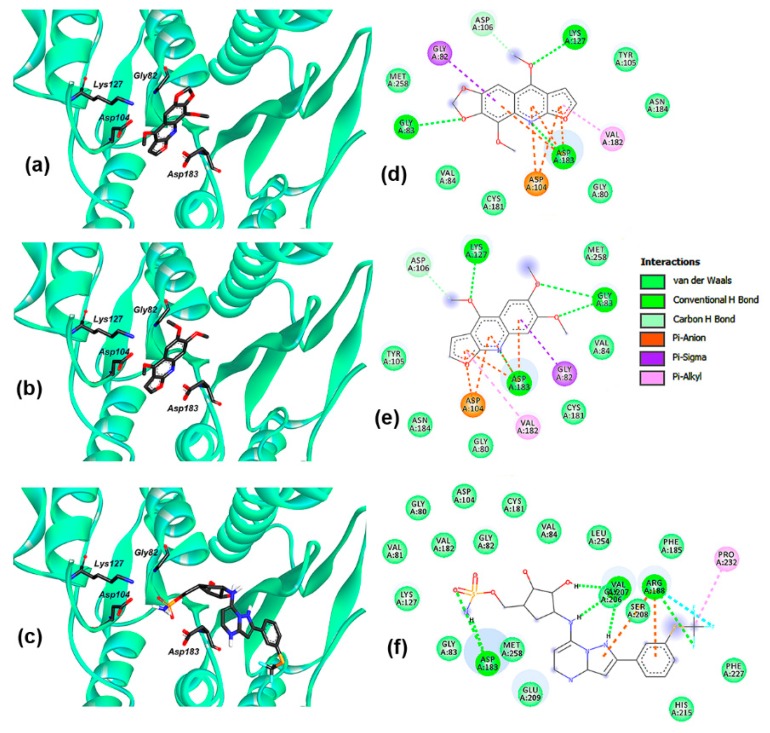
Interaction between test alkaloids **1**–**2** and inhibitor TAK-243 within the binding site of ubiquitin-like modifier-activating enzyme 5 (UBA5). 3D diagrams for (**a**) **1**; (**b**) **2**; (**c**) TAK-243 in complex with UBA5. 2D residual interaction diagrams for (**d**) **1**; (**e**) **2**; (**f**) TAK-243 in complex with UBA5. The different types of interactions between ligand and enzyme residues are exposed with different color according to the interactions panel.

A molecular docking study on **1**–**2** and UBA5 was initially preformed. The resulting docking calculations resulted in good Vina scores (−7.82 ± 0.13 and −7.55 ± 0.16 kcal/mol for **1** and **2**, respectively) in comparison to that of TAK-243 (8.12 ± 0.21 kcal/mol), a known inhibitor for different E1-like Ub-activating enzymes [42]. These results then indicated that alkaloids **1**–**2** might exhibit a reasonable interaction with the binding site of UBA5 (specifically the acidic site owing to the basic nature of alkaloids), but their best-docked poses were found to be located in a different position within this site to that of TAK-243 (Figure 2a–c).

The structural differences between alkaloids **1**–**2** (i.e., 6,7-methylenedioxy and 8-methoxy groups in **1** versus 6,7-dimethoxy groups in **2**) appeared to be non-significant for UBA5 inhibition, since the two-dimensional residual interaction diagrams (Figure 2d,e) showed that both test alkaloids exhibited similar binding modes within the active site of UBA5. Such a binding mode included *H*-bond accepting interactions of 6,7-dimethoxy/6,7-methylendioxy oxygens, 4-methoxy oxygen, and quinoline nitrogen with LYS127, GLY83, and ASP183, respectively, but also showed hydrophobic interactions of aromatic rings, such as Pi-anion, Pi-sigma, Pi-alkyl, with ASP104, GLY82, and VAL182, respectively. These interactions might explain a plausible stabilization of the UBA5:**1** and UBA5:**2** complexes. ASP183 was found to be a common residue involving polar interaction with **1**–**2** and TAK-243, but this inhibitor involved interaction with other residues, specifically within the ATP pocket of UBA5, such as ARG188, VAL207, and PRO232 (Figure 2f) [29]. These residues are in agreement to those of UBA5 bound to ATP [43] and other non-hydrolyzable ATP analogs, such as adenylyl-imidodiphosphate (AMP-PNP), the co-crystalized ligand in the test UBA5 crystal enzyme (PDB code 3GUC), which also rationalizes/justifies our docking simulations.

**Figure 3:**
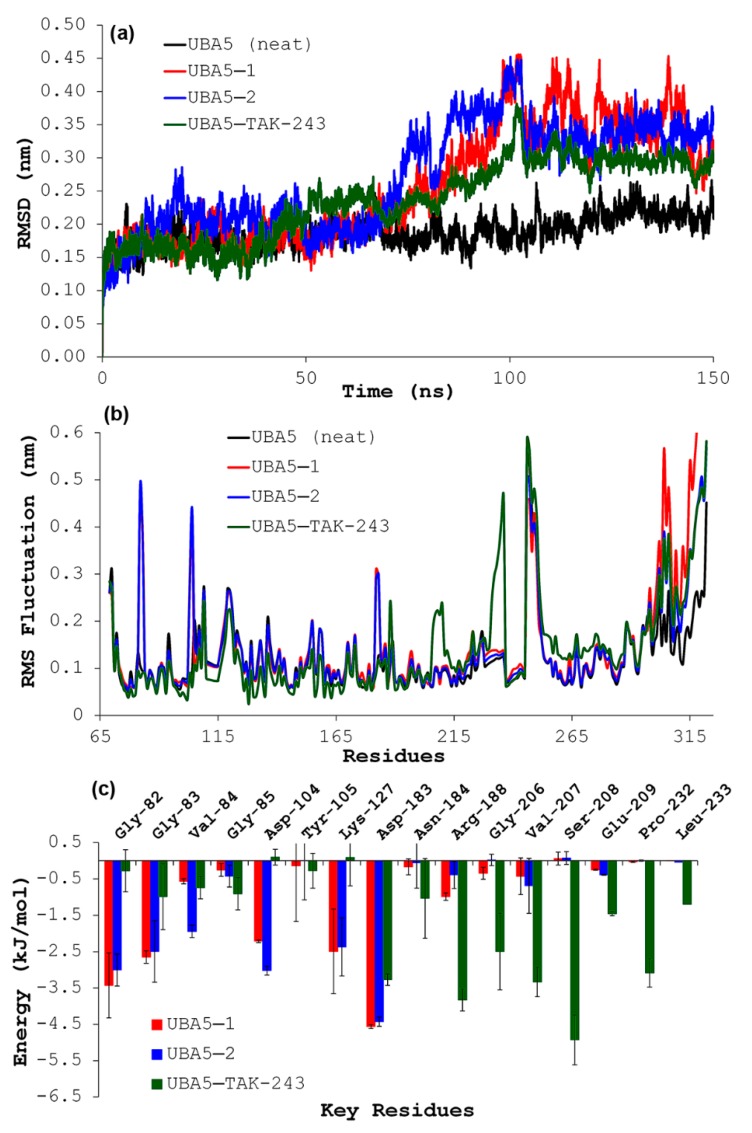
Results after molecular dynamics simulations on the interaction of test alkaloids **1**–**2** and inhibitor TAK-243 with UBA5. (**a**) Cα root-mean-square deviation (RMSD) values (nm) over the simulation time (150 ns) of UBA5 (neat) (black) and complexes UBA5—**1** (red), UBA5—**2** (blue), and UBA5—TAK-243 (green). (**b**) RMS fluctuations of the backbone atoms of UBA5 (black) and complexes UBA5—**1** (red), UBA5—**2** (blue), and UBA5—TAK-243 (green). (**c**) Molecular mechanic/Poisson-Boltzmann surface area (MM/PBSA) per-residue decomposition energies for selected residues of complexes UBA5—**1** (red), UBA5—**2** (blue), and UBA5—TAK-243 (green) after simulation using the last 50 ns.

The in-silico study of the interaction between **1**–**2** and UBA5 was extended using molecular dynamics (MD) simulations as the next step to expand the information about the binding mode of these test alkaloids. Thus, the ligand:enzyme trajectories for those complexes between UBA5 and **1**–**2** and TAK-243 were monitored by means of geometric properties over the time. In this regard, the root mean square deviations (RMSD) were used to evaluate the structural stability of the receptor frame by measuring the time-dependent distance between different positions (in Å) of the set of atoms (Figure 3a).

Hence, separately, 150-ns MD simulations for the UBA5 alone (apoenzyme) and distinctly docked with **1**, **2** and TAK-243 were then recorded for comparison purposes. As result, the UBA5 apoenzyme exhibited a normal evolution during the simulation and a good stabilization after 20 ns (RMSD within 0.15–0.25 nm). The behavior of UBA5–ligand complexes was found to be different to that of the UBA5 apoenzyme, since the structural stabilization seems to be a two-step process. The UBA5–TAK-243 complex reached an initial stabilization step between 5 and 42 ns (0.15–0.20 nm) and, from this point, the complex changed its geometric properties until RMSD values of 0.30–0.35 nm. This complex stabilized after 100 ns (0.25–0.30 nm). In the case of UBA5–**1** and UBA5–**2** complexes, an early stabilization was also achieved (0.15–0.25 nm) between 10 and 65 ns, and a fluctuation was experienced for both complexes until RMSD values of 0.35–0.40 nm at 75 ns. This second stabilization was then maintained over the remaining simulation. Although both alkaloids exhibited a similar RMSD profile, compound **2** promoted fewer perturbations to those of **1**. The analysis of such fluctuations of the atomic positions for each enzyme residue was extended by measurements of the root mean square fluctuation (RMSF), in order to scrutinize the flexibility and secondary structure of the UBA5 enzyme under interaction with the test ligands. The restricted mobility of some residues was then evidenced in apoenzyme and when each ligand was separately introduced to UBA5, while mobility for other residues was slightly higher. All examined complexes showed different behavior (Figure 3b), with explicit differences in some regions, fluctuating significantly along the MD simulations trajectory between RMSF values of 0.04 and 0.55 nm. This fact revealed conformational changes into the enzyme structure in different extents during its interaction with ligands. In this sense, the UBA5–TAK-243 complex exhibited a different RMSF profile to those of UBA5–**1** and UBA5–**2** complexes, specifically through fluctuations by some particular interacting residues. However, complexes involving **1** and **2** showed similar fluctuations. In the specific case of UBA5–**2** complex, fluctuations were observed for the previously identified crucial contacts (i.e., LYS127, GLY83, ASP183, ASP104, GLY82, and VAL182), so its inhibition mode may be achieved by system stabilization through a different manner to that of TAK-243. These results indicated a reasonable in-silico interaction performance of **1**–**2** with the UBA5 enzyme over the time.

The binding free energy for test ligands during interaction with UBA5 for the last 50 ns of MD trajectory was estimated by MM/PBSA approach, after the MD simulations (Table 2). All three ligands exhibited negative binding energies, but alkaloid **2** showed the lower binding energy (−114.5 ± 4.1 kJ/mol) to that of **1** (−102.9 ± 5.7 kJ/mol, respectively) and TAK-243 (−71.6 ± 7.8 kJ/mol). These results might rationalize the observed best docking performance and the best experimental cytotoxic activity of **2** on K562 line. The main contribution to the binding energy of **1**–**2** was due to vdW energies (> −145 kJ/mol). 

These results suggested that weak polar electrostatic interactions are the main driving force for molecular recognition of UBA5 by these alkaloids, specifically the acidic site proximal to the ATP pocket. The above-mentioned fluctuations for each residue were confirmed after binding energy decomposition for the residues of UBA5–ligand complexes (Figure 3c) and different residues were then found to contribute to the interaction energy, depending on ligand (i.e., alkaloids **1**–**2** or TAK-243), as mentioned above. For UBA5–TAK-243 complex, the most binding energy contributing residues were found to be SER208 (−4.9 kJ/mol), ARG188 (−3.8 kJ/mol), VAL207 (−3.3 kJ/mol), and PRO232 (−3.1 kJ/mol), whereas for UBA5–**1** and –**2**, complexes were GLY82, GLY83, ASP104, and LYS127 (see Table 2). ASP183 was the common residue that contributed to the binding energy for both alkaloids **1-2** and TAK-243 to UBA5.

## 4. Conclusions

After a bio-guided fractionation was performed on the ethanol extract from leaves of *E. alata*, the alkaloids kokusaginine **1** and flindersiamine **2** were found to be the main cytotoxic agents on six cancer cell lines at different levels, since these alkaloids retained the cytotoxic activity to that of their parent ethanol extract and raw fraction. As alkaloids showed selectivity to the K562 cell line and lowest cytotoxicity against normal human lung fibroblasts, these alkaloids can be proposed as leads for further studies against myelogenous leukemia. In this regard, the in-silico study indicated that alkaloids **1**–**2** exhibited good interaction profile with human ubiquitin-activating enzyme 5 (UBA5), involving weak polar electrostatic interactions for the recognition of the acidic site of UBA5 proximal to the ATP pocket by these alkaloids due to basic nature, so they could be considered potential leads for the future development of UBA5 inhibitors based on furoquinolines. As computational hypotheses related to the insights into the interaction, these alkaloids showed a particularly different binding mode to that of a known UAE E1 inhibitor (i.e., TAK-243). Such a mode of action through UBA5 inhibition will be further explored by in-vitro enzymatic inhibition over purified enzyme. Thus, after data analyses, some important hits and structural requirements were established, indicating the potential of furoquinoline alkaloids for disrupting cancer progression by ubiquitin–proteasome pathway perturbation or acting synergistically with kinase inhibitors to induce apoptosis of K562 cells. Hence, this information can be preserved for further studies focused on the development of antitumor agents based on furoquinoline alkaloids.

## Figures and Tables

**Table 1:** Cytotoxic activity of *E. alata* leaves-derived ethanol extract, AcOEt fraction, and alkaloids **1**–**2**.

Treatment	Cell Inhibition (%)						
	Cancer Cell Lines *^g^*						
	U251	PC-3	K562	HCT-15	MCF-7	SKLU-1	MRC5
**EEa *^a^***	29.5 ± 2.5	68.3 ± 1.2	96.8 ± 1.2	43.5 ± 2.7	62.7 ± 1.9	66.5 ± 4.5	18.3 ± 2.1
**CEa *^b^***	27.4 ± 3.1	67.9 ± 1.5	97.2 ± 0.9	48.7 ± 2.1	45.7 ± 2.4	60.3 ± 3.1	21.3 ± 1.8
**EA-EEa3 *^c^***	35.3 ± 2.7	61.4 ± 1.9	88.3 ± 1.8	44.5 ± 2.7	48.4 ± 3.3	31.5 ± 2.8	11.4 ± 1.2
**EA-EEa6 *^d^***	30.2 ± 4.2	63.2 ± 2.2	91.7 ± 1.4	46.2 ± 1.2	36.5 ± 1.7	25.6 ± 3.2	19.9 ± 1.5
**1 *^e^***	31.9 ± 1.8	58.5 ± 2.0	86.7 ± 1.8	58.0 ± 3.2	31.9 ± 2.3	32.7 ± 1.7	5.3 ± 0.9
**2 *^e^***	43.4 ± 2.1	59.8 ± 1.7	93.4 ± 2.3	66.5 ± 2.5	52.8 ± 2.9	30.8 ± 2.2	4.3 ± 1.1
**doxorubicin *^f^***	98.7 ± 0.9	82.1 ± 1.8	91.4 ± 1.9	93.2 ± 2.5	87.0 ± 3.1	90.8 ± 2.8	77.4 ± 3.2

*^a^ E. alata* leaves-derived ethanol extract (50 µg/mL); *^b^* AcOEt fraction after CCV fractionation (30 µg/mL); *^c^* Subfraction obtained from AcOEt containing alkaloid **2** (10 µg/mL); *^d^* Subfraction obtained from AcOEt containing alkaloid **1** (10 µg/mL); *^e^* Isolated alkaloids **1**–**2** (20 µM); *^f^* Positive control (10 μM); *^g^* Cancer cell lines: U251 (malignant glioblastoma), PC-3 (prostate carcinoma), K562 (myelogenous leukemia), HCT-15 (colon carcinoma), MCF-7 (breast adenocarcinoma), and SKLU-1 (lung adenocarcinoma), and MCR5 (normal human lung fibroblasts).

**Table 2:** Binding energies (kJ/mol) for alkaloids **1**–**2** and TAK-243 in complex with ubiquitin-like modifier-activating enzyme 5 (UBA5) using molecular mechanic/Poisson-Boltzmann surface area (MM/PBSA) method among 100–150 ns of molecular dynamics simulations.

Complex	E_vdw_	E_elec_	G_polar_	G_nonpolar_	ΔG_binding_
UBA5—**1**	−145.3 ± 5.1	−17.8 ± 3.8	75.6 ± 7.5	−15.4 ± 0.5	−102.9 ± 5.7
UBA5—**2**	−156.2 ± 7.9	−16.3 ± 7.1	73.9 ± 17.2	−15.9 ± 0.5	−114.5 ± 4.1
UBA5—TAK-243	−186.7 ± 6.3	−88.4 ± 12.6	222.7 ± 7.2	−19.2 ± 0.6	−71.6 ± 7.8

## Supplementary Materials

The following are available online at https://www.mdpi.com/2218-273X/9/10/585/s1, Figures S1–S4. ^1^H-NMR (400 MHz, CDCl_3_) and ^13^C-NMR (100 MHz, CDCl_3_) spectra of compounds **1**–**2**.
