# Supplementary material for: Bio-Guided Fractionation of Ethanol Extract of Leaves of Esenbeckia alata Kunt (Rutaceae) Led to the Isolation of Two Cytotoxic Quinoline Alkaloids: Evidence of Selectivity Against Leukemia Cells
Source: Biomolecules. 2019 Oct 8;9(10):585. doi: 10.3390/biom9100585 (PMC6843300; doi:10.3390/biom9100585)

Supplementary Material

Bio-Guided Fractionation of Ethanol Extract of Leaves of *Esenbeckia alata* Kunt (Rutaceae) Led to the Isolation of Two Cytotoxic Quinoline Alkaloids: Evidence of Selectivity Against Leukemia Cells

Juan Manuel Álvarez-Caballero [1*], Luis Enrique Cuca-Suárez [2], Ericsson Coy-Barrera [3]

[1] Grupo de Química y Bioprospección de Productos Naturales, Universidad del Magdalena, Santa Marta, Magdalena, Colombia.
[2] Laboratorio de Investigación en Productos Naturales Vegetales, Facultad de Ciencias, Departamento de Química, Universidad Nacional de Colombia, Bogotá D.C., Colombia.
[3] Bioorganic Chemistry Laboratory, Facultad de Ciencias Básicas y Aplicadas, Universidad Militar Nueva Granada, Cajicá 250247, Colombia.

Content

S1. ^{1}H NMR of compound 1 (400 MHz, $CDCl_3$) 2

S2. ^{13}C NMR of compound 1 (100 MHz, $CDCl_3$) 2

S3. ^{1}H NMR of compound 2 (400 MHz, $CDCl_3$) 3

S4. ^{13}C NMR of compound 2 (100 MHz, $CDCl_3$) 3

S1. ^{1}H NMR of compound 1 (400 MHz, CDCl₃)

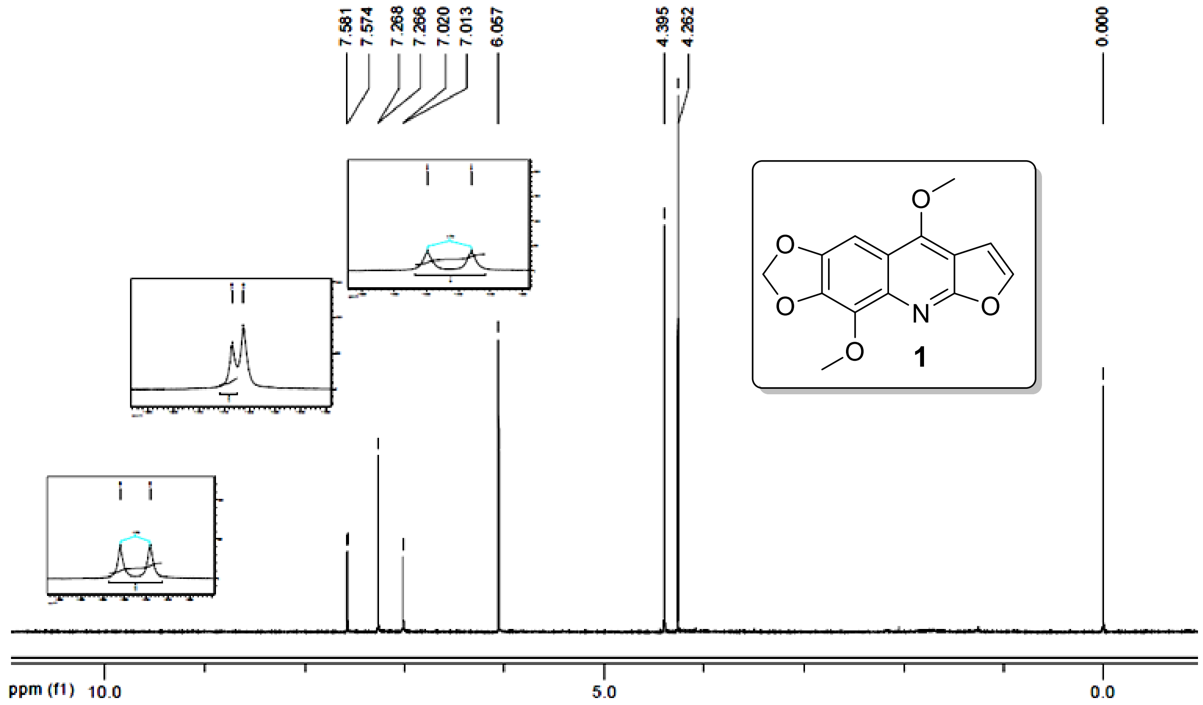

S2. ^{13}C NMR of compound 1 (100 MHz, CDCl₃)

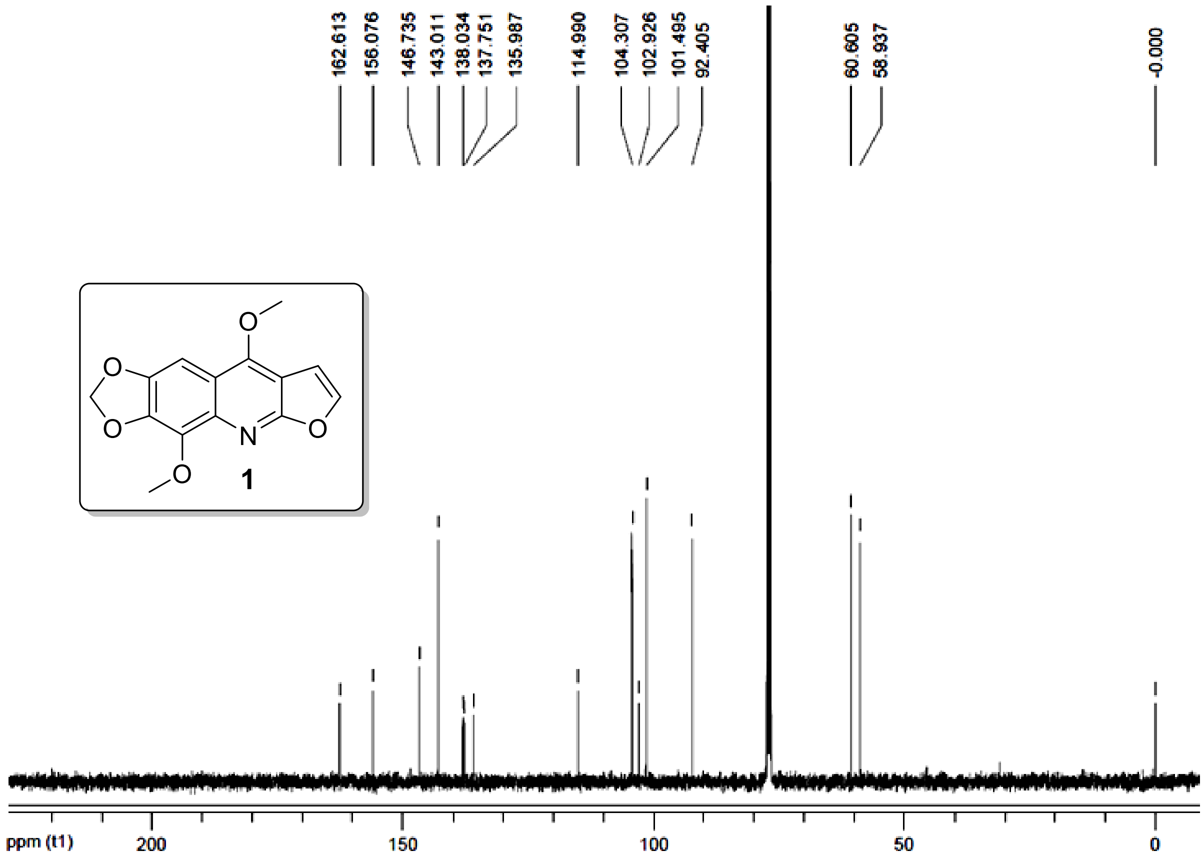

S3. ^{1}H NMR of compound 2 (400 MHz, $CDCl_3$)

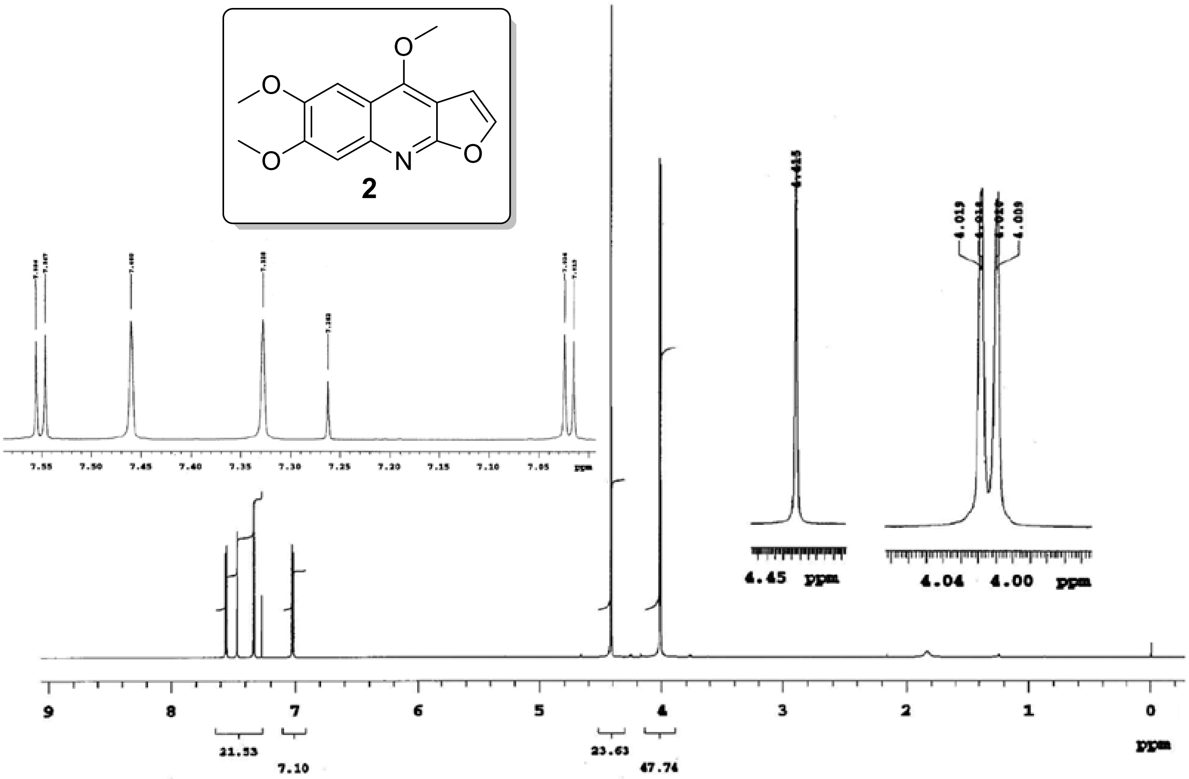

S4. ^{13}C NMR of compound 2 (100 MHz, $CDCl_3$)

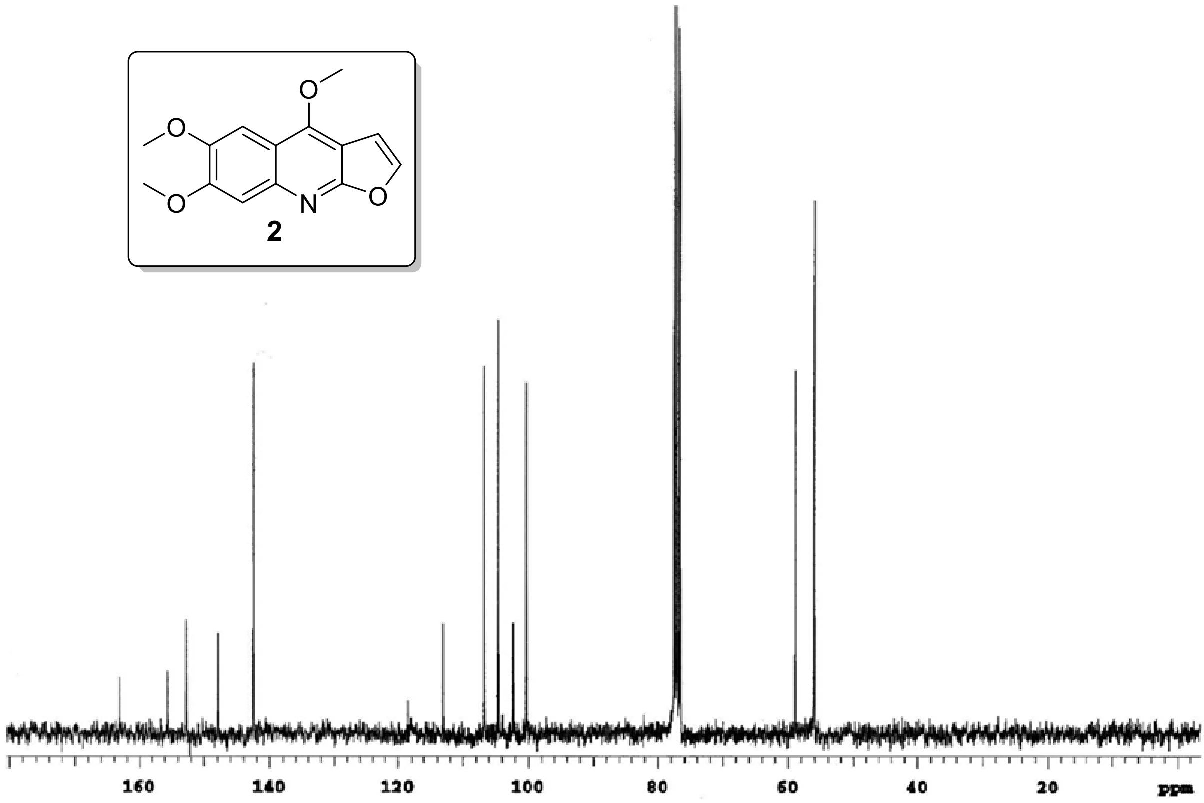

Supplement: Supplementary file 1 [file biomolecules-09-00585-s001.pdf]
